# Visceral leishmaniasis and HIV/AIDS in Brazil: Are we aware enough?

**DOI:** 10.1371/journal.pntd.0005772

**Published:** 2017-09-25

**Authors:** Marcia Leite de Sousa-Gomes, Gustavo Adolfo Sierra Romero, Guilherme Loureiro Werneck

**Affiliations:** 1 Secretary of Health Surveillance, Ministry of Health, Brasilia, Distrito Federal, Brazil; 2 Professional Master Program in Epidemiology in Public Health, National School of Public Health, Oswaldo Cruz Foundation, Rio de Janeiro, Rio de Janeiro, Brazil; 3 Center for Tropical Medicine, University of Brasilia, Brasilia, Distrito Federal, Brazil; 4 National Institute for Science and Technology for Health Technology Assessment (IATS/CNPq), Porto Alegre, Rio Grande do Sul, Brazil; 5 Institute of Social Medicine, Rio de Janeiro State University, Rio de Janeiro, Rio de Janeiro, Brazil; Istituto Superiore di Sanità, ITALY

## Abstract

**Background:**

The urbanization of visceral leishmaniasis (VL) and the concurrent movement of the HIV infection to rural areas in Brazil are possible mechanisms associated with an increased number of *Leishmania*/HIV coinfected people. This study aimed to describe the clinical and epidemiological profile of VL/HIV coinfected patients and compare this profile to non-coinfected VL patients.

**Methods:**

Cases of VL/HIV coinfection were obtained through a probabilistic record linkage of databases of VL and AIDS cases from the Brazilian Ministry of Health.

**Results:**

We retrieved 760 cases of VL/HIV coinfection, most prevalent in adult males, with incidence ranging from 0.01 to 0.07 cases, per 100.000 population, in 2001 and 2010, respectively. Case-fatality rates were 27.3% in 2001 and 23.2% in 2010. Weakness, weight loss, cough, other associated infections and haemorrhagic phenomena were more commonly found among coinfected patients, which had a fatality rate three times higher as compared to the non-coinfected group. The relapse proportion was two times greater among coinfected (6.3%) than non-coinfected (3.1%).

**Conclusions:**

The results found herein contribute to the increase of knowledge of the epidemiological situation of VL/HIV coinfection in Brazil and reinforce the necessity of implementing specific strategies to improve early case detection and efficacious and less toxic treatment in order to achieve lower case-fatality rates.

## Author summary

Visceral leishmaniasis (VL) is an infectious disease present in 75 countries. Brazil is among the six countries that represent over 90% of the cases worldwide. AIDS is an emerging disease present in approximately 190 countries and a major public health problem worldwide due to its magnitude and damage extension. In Brazil, the recent alterations of the AIDS and VL distribution patterns, such as movement of the HIV infection to rural areas and the urbanization of VL associated to the rise of VL cases among the 20–49 years old age group, are possible factors associated with an increased risk of the population to present both infections. The coexistence of these diseases enhances the severity, seeing that VL accelerates the development of AIDS in HIV patients, and AIDS increases the risk of VL up to 100 to 1,000 times in endemic areas. Considering that the association of both infections is recent, however rising in several countries, our study contributes to the knowledge of the profile, magnitude and severity of VL/HIV coinfection, which can subsidize actions to overcome challenges related to both VL and AIDS and assist in guiding actions and decision-making processes.

## Introduction

Visceral leishmaniasis (VL) is an anthropozoonosis associated to infection by different protozoa species of the genus *Leishmania*, primarily transmitted by the bite of infected phlebotomine sandflies. In Brazil, the disease is caused by *Leishmania infantum*, transmitted by vectors of the genus *Lutzomyia* [1, 2], with an estimated annual incidence of 4,200 to 6,300 cases and a case-fatality rate of 7% [3, 4].

Infection by the human immunodeficiency virus (HIV) is a severe worldwide public health problem, with an estimate of 36.9 million people living with HIV infection and 2 million new infections occurring each year. Although HIV infection is widespread, the burden of the disease is much higher in sub-Saharan Africa, followed by Southeast Asia, the Americas, Europe, Western Pacific, and the Eastern Mediterranean region. Estimates indicate that 1/3 of the persons with HIV infection live in risk areas for leishmaniasis transmission. This geographic overlap results in an increasing number of HIV-*Leishmania* coinfected cases which has been reported in 35 countries [5, 6].

The severity of the VL-HIV coinfection is worse in some African countries, such as the Sudan and Ethiopia, where 35% of all VL patients are also infected by HIV [7]. In Brazil, the recent alterations of the acquired immunodeficiency syndrome (AIDS) and VL distribution patterns, such as movement of the HIV infection to rural areas and the urbanization of VL associated to the rise of VL cases among the 20–49 years old age group, have pointed that the population has a more likely risk to present both infections [8, 9]. Despite the Brazilian policy of free access to highly active anti-retroviral therapy (HAART), the country has a relevant number of HIV infected persons who ignore their HIV status leading to delayed first consultation after the appearance of advanced symptomatic immunodeficiency.

The increasing number of VL/HIV coinfected cases in the world contrasts with the European scenario where the introduction of the (HAART) led to a decrease in the number of VL/HIV coinfected cases particularly in Spain, France and Italy [10–12].

Considering the current worldwide epidemiological of VL/HIV infection worldwide, the use of analytical descriptive tools, such as mapping, pattern analysis and identification of population profiles, are essential to the development of strategies to approach the clinical and epidemiological challenges presented by VL/HIV coinfection [13].

The present information available in scientific literature from Brazil is restricted to case reports and a few local analyses of serial cases of VL/HIV coinfection, justifying the development of a broader analysis of the occurrence of this coinfection in the whole country.

This study describes the clinical and epidemiological profile of VL patients coinfected with HIV and compares this profile with that of VL patients without coinfection.

## Methods

### Ethics statement

This Project was appreciated and approved by the Research Ethics Committee of the Sergio Arouca National School of Public Health–CEP/ENSP of the Oswaldo Cruz Foundation (CAAE: 00613312.6.0000.5240). The information used in this study was taken from secondary databases, so no ethics statement was required. All the information that identifies the patient was anonymized.

### Study design

A descriptive and exploratory study was carried out with secondary data of VL and AIDS cases in Brazil. We analyzed data from all 21 states that presented VL cases from 2001 to 2010.

### Population and source of data

The study population was constituted by all VL cases, from 2001–2010, reported to the Notifiable Diseases Information System (Sinan, acronym in Portuguese) after a preliminary analysis to remove duplicities. A single database for AIDS cases was created from the integration of cases reported to Sinan, the Mortality Information System (SIM, acronym in Portuguese), the Laboratory Tests Control System (Siscel, acronym in Portuguese), and the Medication Logistics Control System (Siclom, acronym in Portuguese), from 1980 to June 2011.

### Inclusion criteria

In order to associate the VL and AIDS databases all VL cases reported to Sinan were included in this study. For analyses purposes, we considered only VL Sinan confirmed cases or cases with at least one record of positive result by some VL laboratory diagnostic test. During the study period, the Brazilian Ministry of Health (MoH) recommended that all VL suspicious cases were to be notified and investigated through a standardized information form available at the Sinan. Given the relevance of HIV coinfection in the clinical progression of this disease, there is a specific field in this form to register this information; however only approximately 65% of the forms registered such data. This fact justified the search for coinfection cases not registered in the VL database at Sinan through the association of this system to other information systems with records of AIDS cases and deaths in Brazil.

Throughout the research period, notification of HIV asymptomatic infection, which did not require specific treatment, was not mandatory in the country. Thus, all HIV patients registered in the database for AIDS cases presented AIDS symptoms or were considered as people living with the disease, with the need of some sort of specific therapeutic intervention to prevent disease progression.

### Data processing and analysis

Considering that the VL and AIDS database had no univocal identifier, the RecLink III [14] software was used as a method for database linkage, implementing a probabilistic record linkage. As suggested by Camargo Jr & Coeli [14], in this study we considered the following variables for matching records from the different databases: name of the patient, name of the mother and date of birth.

The multiple steps strategy was used for the blocking, noting that in each stage only not previously paired records were evaluated [15]. We opted to carry out three consecutive stages: 1^st^) *soundex* codes of the first and last name of the patient, taking into account if the names had identical sounds, disregarding the spelling, in combination with the gender and municipality of residence; 2^nd^) *soundex* codes of the first and last name of the patient in combination with gender, and 3^rd^) *soundex* codes of the first and last name of the patient.

A score value, corresponding to each pairing, was generated after the blocking stage indicating the true value of each identified pair. Pairs with a minimum score value equal to 19 were considered as true pairs, and score values below 10 were considered as non-pairs. We performed a manual inspection of the doubtful areas (“grey area”) between score values of 10 to 19, in search for other possible pairs. At the end of this process, records not considered as pairs were excluded from the final archive.

Lastly, after the three blocking stages and the manual inspection, the true pairs archives were combined into one single archive, which was manually inspected to remove eventual duplicated pairs.

From this database, the absolute number of cases was considered to estimate the magnitude of the VL/AIDS coinfection in Brazil. Furthermore, we calculated the proportion of coinfected cases, out of the total of VL confirmed cases, the incidence rate per 100,000 population, and the fatality rate. The population data was obtained from the Brazilian Institute of Geography and Statistics (IBGE, acronym in Portuguese) [16].

The spatial distribution of the VL/AIDS coinfected patients was assessed by thematic maps produced with the Terraview software (4.2.0 version), according to the state and municipality of residence.

To generate a map, with the areas with greater risk of VL/HIV coinfection, the average incidence per 100,000 population was calculated. Where, the numerator was composed by the average number of incident confirmed cases, by state of residence along the study period, and the denominator by the population data of the IBGE in the middle of the study period (07.01.2006).

Afterwards, the VL cases were categorized into three groups for clinical-epidemiological comparative analysis purposes:

- Group 1 –VL/AIDS Coinfected patients: individuals that, after the probabilistic linkage procedure, were found in both VL and AIDS databases, regardless of the filling of the HIV specific field (positive, negative, ignored) of the VL information form;
- Group 2 –VL/HIV Coinfected patients: individuals that, after the probabilistic linkage procedure, were not found in the AIDS database; however, were marked in the VL database as “positive” in the HIV specific field of the VL information form;
- Group 3 –Non-coinfected patients: individuals that, after the probabilistic linkage procedure, were not found in the AIDS database; however, were marked in the VL database as “negative” or as “ignored” in the HIV specific field of the VL information form.

A completeness assessment was performed with the initially proposed variables to select those to be included in the comparative analyses between the groups. The variables with 50% of blank or ignored information were excluded.

Considering the possible influence of the age variable among the other ones, the normality test was done for each group through the Kolmogorov-Smirnov test. Subsequently, the Kruskal-Wallis test was performed to confirm significant statistical differences (p<0.05) in the average age of the three groups. Lastly, the Mann-Whitney test for between-groups comparisons with Bonferroni correction was used to identify possible age differences between each pair of groups.

Initially, comparative analyses were performed separately for two age groups; since, for epidemiological surveillance purposes, the cases definition of the Brazilian Department of STD/AIDS and viral hepatitis are: children under 13 years of age and patients aged 13 years or over [17].

The differences between the groups for the demographic, clinical and epidemiological profiles were analyzed for each of the selected variables through the difference among the proportions of the two groups and their respective 95% confidence intervals.

## Results

Through the association of the databases, we could identify 1,447 possible pairs of VL/AIDS coinfected patients, from the total of notified VL cases, from 2001–2010 (65,914). From these, 760 met the inclusion requirements of this study (Group 1), representing 2.1% of the total of confirmed VL cases (35,819). Out of these 760 coinfected patients, 500 were marked as HIV positive in the VL information sheet, 161 as negative, and 99 as blanks or ignored.

We additionally found that 541 notified cases in the VL database were marked as HIV positive but were not identified in the AIDS database (Group 2). Thus, the total number of VL/HIV coinfected cases identified in this study was 1,301 (Group 1 + Group 2).

**Fig 1:**
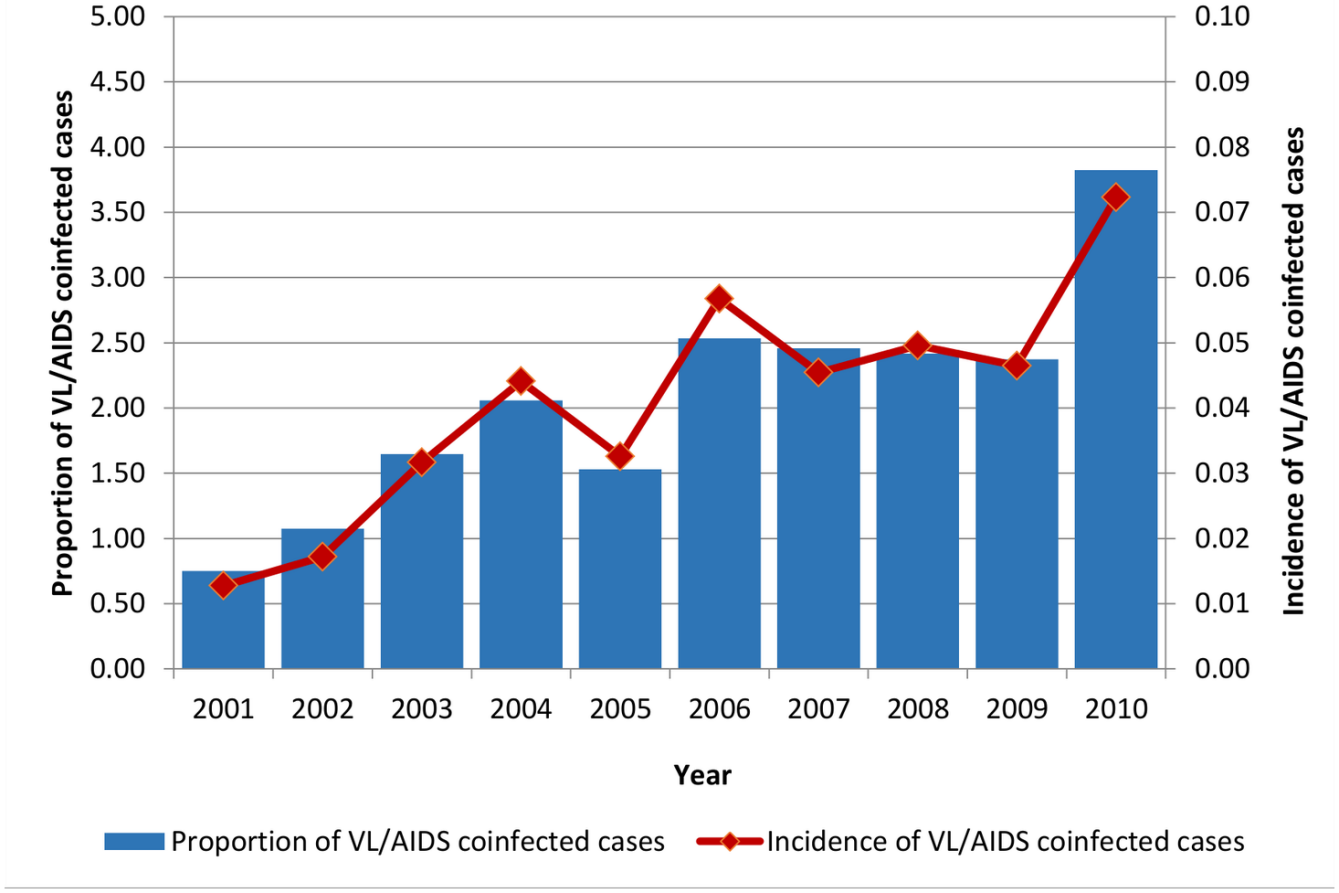
Proportion and incidence of VL/AIDS coinfection cases according to the year of notification. Brazil. 2001–2010. Data adapted from Sinan/SVS/MS, 2012.

Fig 1 shows the proportion of VL cases coinfected with AIDS and the incidence of VL/AIDS coinfection from 2011 to 2010. In 2001, from the total of confirmed VL cases, 0.75% were coinfected with AIDS, increasing to 2.53% in 2006 and, after a stable period until 2009, reached 3.82% in 2010, an approximately five-fold increase over the period. This same pattern can be identified in the incidence rates. In 2010, the incidence was 0.07 cases per 100,000 population, which is seven-fold higher than in 2001 (0.01 cases per 100,000 population) (Fig 1).

**Fig 2:**
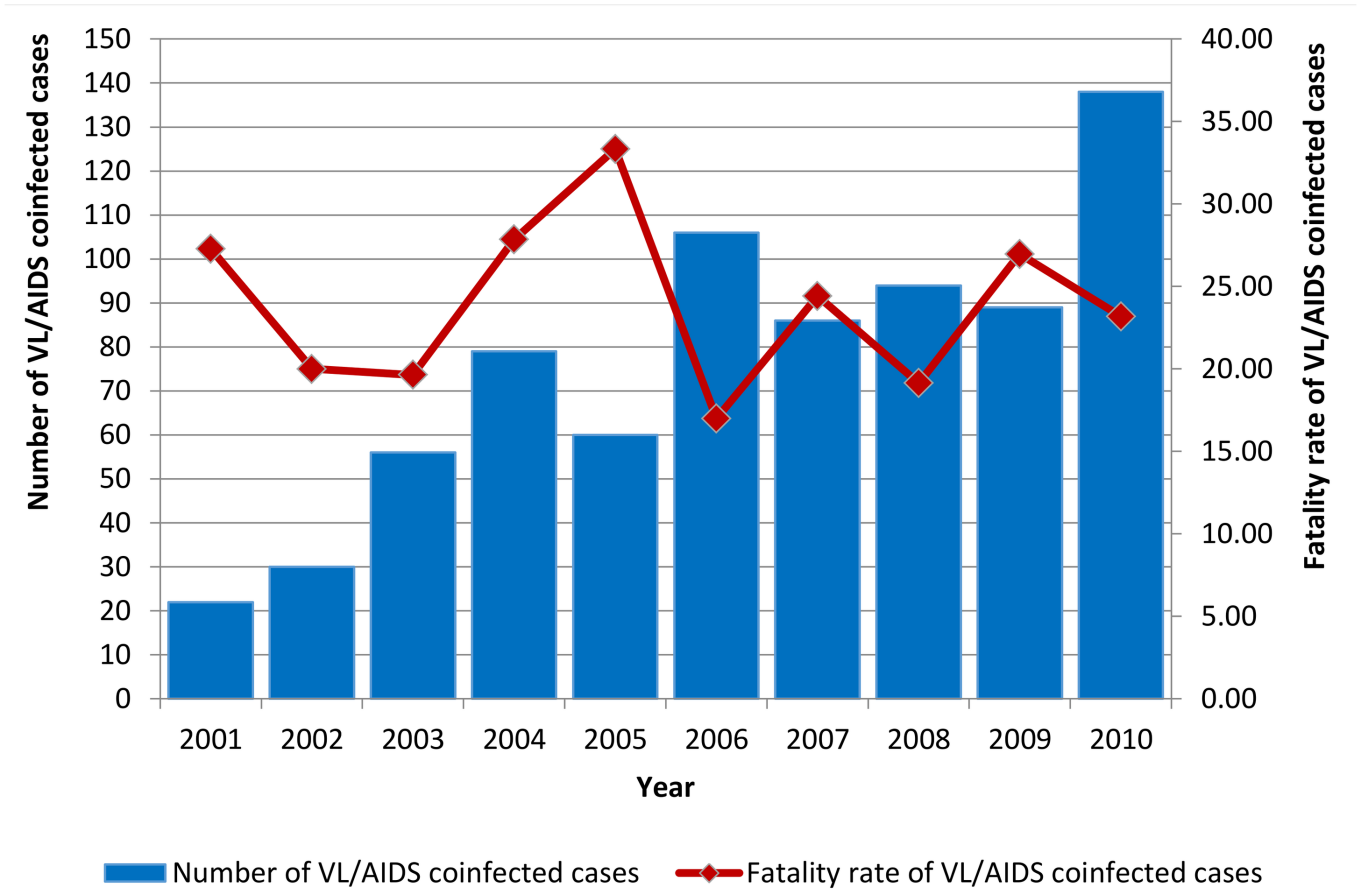
Number of cases and fatality rate of VL/AIDS coinfected patients according to the year of notification. Brazil. 2001–2010. Data adapted from Sinan/SVS/MS, 2012.

Fig 2 shows the number of VL/AIDS cases and associated fatality rates from 2001 to 2010. The number of VL/AIDS cases increased steadily from 2001 to 2004 and, after a decrease in 2005, increased again in 2006, staying stable until a new increase was recorded in 2010. The fatality rates of VL/AIDS coinfected patients showed a less clear trend, being 27.27% in the first year of this study (2001) and 23.19% in 2010, indicating a relative 17% decrease during the period. The highest fatality rate was registered in 2005 (33.33%) and the lowest in the subsequent year (16.98%) (Fig 2).

**Fig 3:**
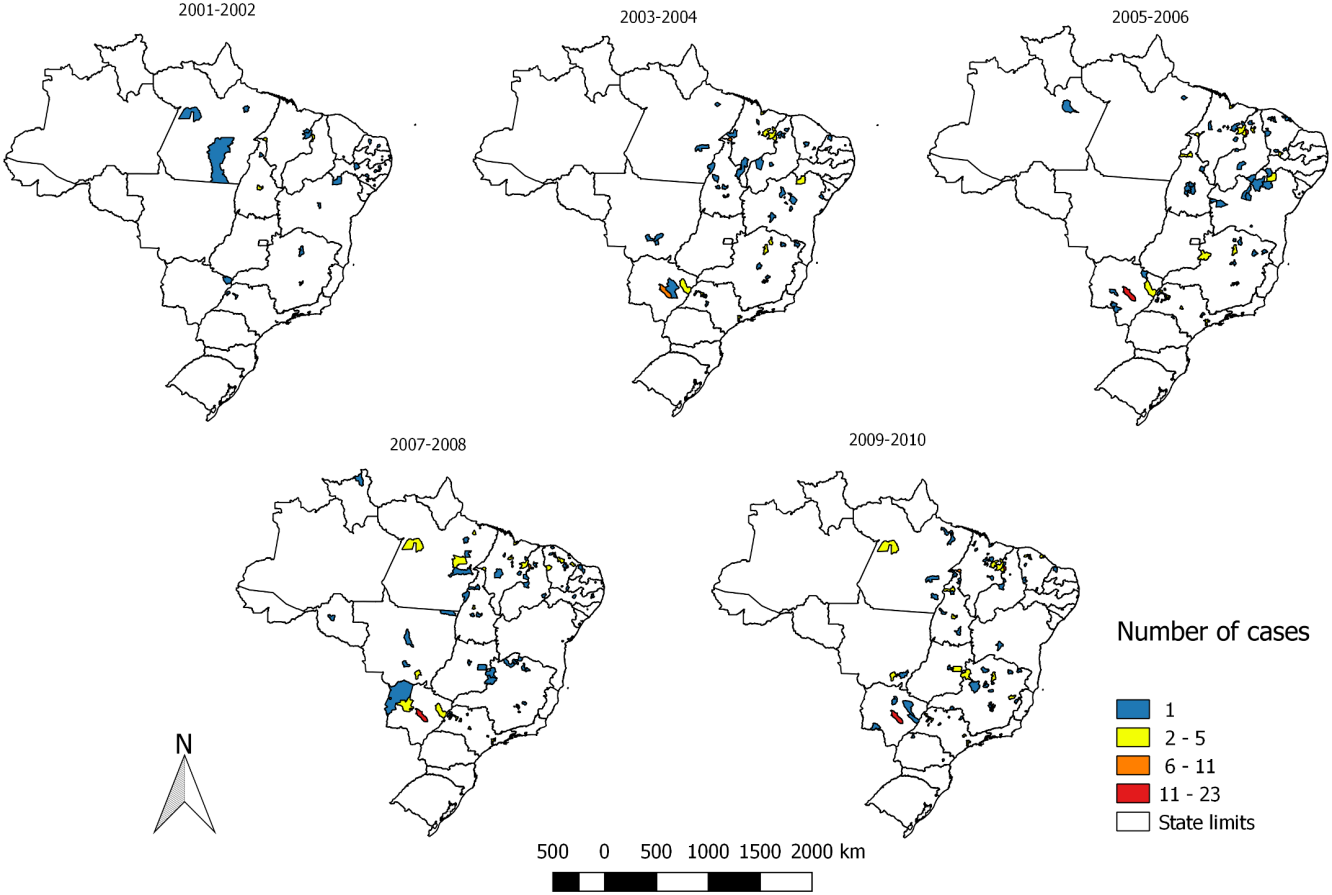
Distribution of the VL/AIDS coinfected cases according to the municipality of residence per biennium. Brazil. 2001–2010. Data adapted from Sinan/SVS/MS, 2012.

From 2001–2002 the VL/AIDS coinfection cases were predominantly distributed in municipalities of the North and Northeast Region of the country. However, along the next periods we noticed an increase in the number of municipalities with this coinfection, reaching mainly the Southeast and Midwest Regions of the country (Fig 3).

From the 35,819 VL cases included in this study, 760 (2.12%) belonged to group 1 (VL/AIDS coinfected patients), 541 (1.51%) to group 2 (VL/HIV coinfected patients) and 34,518 (96.37%) to group 3 (non-coinfected patients).

Considering that children represented a small percentage of the VL/AIDS and VL/HIV coinfected cases (12.14%) and 58% of the non-coinfected VL patients, we opted to perform an initial descriptive analysis separately for two age groups: children under 13 years of age, and patients aged 13 years or over (S1 and S2 Tables, respectively).

The total of children under 13 in this study was 20,233 (56.49%), of which 33 (0.16%) were VL/AIDS coinfected patients (Group 1), 125 (0.62%) were VL/HIV coinfected patients (Group 2) and 20,075 (99.2%) were non-coinfected (Group 3). In general, VL/AIDS children were older and more frequently from urban areas than their VL/HIV coinfected and non-coinfected counterparts. The male:female ratio was also higher among VL/AIDS children. Both VL/AIDS and VL/HIV children were more likely to present hemorrhagic phenomena and to progress to death than the non-coinfected group. Such differences should be considered with caution because of the small numbers of coinfected children, particularly in the VL/AIDS group. A detailed demographic, clinical and epidemiological characterization of this age group, according to the three analyzed groups, is described in S1 Table.

The total of patients aged 13 years or over in this study was 15,586 (43.51%), of which 727 (4.66%) were VL/AIDS coinfected patients (Group 1), 416 (2.67%) were VL/HIV coinfected patients (Group 2) and 14,443 (92.67%) were non-coinfected (Group 3). In general, there were not major differences between Groups 1 (VL/AIDS) and 2 (VL/HIV), although the first progressed to death more frequently (28%) than the last (21.7%). However, both Groups 1 and 2 showed substantial differences in relation to Group 3 (non-coinfected). Groups 1 and 2 more frequently came from urban areas, presented infections, had a positive parasitological diagnosis, were diagnosed as relapse cases, used amphotericin and evolved to death. A detailed demographic, clinical and epidemiological characterization of this age group, according to the three analyzed groups, is described in S2 Table.

For both children under 13 years of age and patients aged 13 years or over the descriptive analyses revealed a resemblance of the distribution of the variables between groups 1 (VL/AIDS coinfected) and 2 (HIV/VL coinfected) (S1 and S2 Tables). Considering that the individual management of the HIV-infected patient in the presence of VL is similar to the patient with AIDS and VL, [18] we decided to merge groups 1 and 2 in a single group for the subsequent analyses of differences between the coinfected and non-coinfected profiles, as described in Table 1.

**Table 1:** Demographic, clinical and epidemiological characteristics of coinfected and non-coinfected cases. Brazil. 2001–2010.

Characteristics	Coinfected			Non-coinfected			Difference between proportions^2^	
	Frequency (total)	%	95%CI	Frequency (total)	%	95%CI	%	95%CI
**Age**								
** average**	34.07		33.20,34.94	18.73		18.49,18.97	-15.34	-16.60,-14.08
** median**	35			7				
** standard deviation**	16.03			23.05				
**Gender**								
** male**	973 (1301)	74.8	72.4,77.1	20878 (34515)	60.5	60.0,61.0	-14,3	-16.7,-11.9
** female**	328 (1301)	25.2	22.9,27.6	13637 (34515)	39.5	39.0,40.0	14.3	11.9,16.7
**Residency area**								
** urban/peri-urban**	1096 (1250)	87.7	85.7,89.4	23642 (33400)	70.8	70.3,71.3	-16.9	-18.8,-15.0
** rural**	154 (1250)	12.3	10.6,14.3	9758 (33400)	29.2	28.7,29.7	16.9	15.0,18.8
**Clinical manifestation**								
** fever**	1151 (1252)	91.9	90.3,93.3	31547 (32980)	95.7	95.4,95.9	3.8	2.3,5.4
** weakness**	1051 (1203)	87.4	85.4,89.1	25125 (30468)	82.5	82.0,82.9	-4.9	-6.7,-2.9
** weight loss**	1041 (1215)	85.7	83.6,87.5	23294 (30553)	76.2	75.8,76.7	-9.5	-11.4,-7.3
** cough**	710 (1178)	60.3	57.5,63.0	14801 (29804)	49.7	49.1,50.2	-10.6	-13.4,-7.7
** splenomegaly**	951 (1193)	79.7	77.3,81.9	27940 (32330)	86.4	86.0,86.8	6.7	4.5,9.1
** hepatomegaly**	864 (1185)	72.9	70.3,75.4	24419 (31920)	76.5	76.0,77.0	3.6	1.1,6.2
** edema**^1^	172 (674)	25.5	22.4,28.9	3155 (12467)	25.3	24.5,26.1	-0.2	-3.7,3.0
** pallor**^1^	511 (685)	74.6	71.2,77.7	9564 (12663)	75.5	74.8,76.3	0.9	-2.3,4.4
** infectious process**^1^	266 (660)	40.3	36.6,44.1	2896 (12109)	23.9	23.2,24.7	-16.4	-20.2,-12.6
** hemorrhagic phenomena**^1^	105 (664)	15.8	13.2,18.8	1246 (12367)	10.1	9.6,10.6	-5.7	-8.8,-3.1
** jaundice**^1^	130 (660)	19.7	16.8,22.9	3020 (12401)	24.4	23.6,25.1	4.7	1.4,7.6
**Parasitological diagnosis**								
** positive**	689 (831)	82.9	80.2,85.3	13735 (17151)	80.1	79.5,80.7	-2.8	-5.3,-0.1
** negative**	142 (831)	17.1	14.7,19.8	3416 (17151)	19.9	19.3,20.5	2.8	0.1,5.3
**Immunological diagnosis (IFA)**								
** positive**	484 (607)	79.7	76.4,82.7	14624 (16903)	86.5	86.0,87.0	6.8	3.7,10.2
** negative**	123 (607)	20.3	17.3,23.6	2279 (16903)	13.5	13.0,14.0	-6.8	-10.2,-3.7
**Patient entry**								
** new case**	1153 (1230)	93.7	92.2,95.0	31640 (32636)	96.9	96.8,97.1	3.2	2.0,4.7
** relapse**	77 (1230)	6.3	5.0,7.7	996 (32636)	3.1	2.9,3.2	-3.2	-4.7,-2.0
**Initial drug administrated**								
** pentavalent antimony**	727 (1117)	65.1	62.2,67.8	26248 (30490)	86.1	85.7,86.5	21.0	18.2,23.9
** amphotericin b**	297 (1117)	26.6	24.1,29.3	2211 (30490)	7.3	7.0,7.5	-19.3	-21.9,-16.7
** pentamidine**	3 (1117)	0.2	0.0,0.7	143 (30490)	0.4	0.4,0.6	0.2	-0.1,0.5
** other**	34 (1117)	3.0	2.1,4.2	880 (30490)	2.9	2.7,3.1	-0.1	-1.1,0.9
** not used**	56 (1117)	5.0	3.9,6.5	1008 (30490)	3.3	3.1,3.5	-1.7	-3.0,-0.4
** liposomal amphotericin b**	100 (697)	14.3	11.9,17.1	854 (12512)	6.8	6.4,7.3	-7.5	-10.2,-4.9
**Progression**								
** recovery**	804 (1072)	75.0	72.3,77.5	26007 (28358)	91.7	91.4,92.0	16.7	14.2,19.4
** death**	268 (1072)	25.0	22.5,27.7	2351 (28358)	8.3	8.0,8.6	-16.7	-19.4,-14.2
**Confirmation criterion**^1^								
** laboratory**	657 (747)	87.9	85.4,90.1	11771 (13847)	85.0	84.4,85.6	-2.9	-5.2,-0.3
** clinical epidemiological**	90 (747)	12.1	9.9,14.6	2076 (13847)	15.0	14.4,15.6	2.9	0.3,5.2

^1^ Variables available in the VL database from 2007–2010.

^2^ Difference between proportions, except for the age corresponding to the average difference.

As depicted in Table 1, differences between coinfected and non-coinfected were detected for all characteristics evaluated. In comparison to non-coinfected cases, the coinfected patients were older, predominantly male and from urban/peri-urban areas. Regarding VL clinical manifestations coinfected patients showed a higher frequency of weakness, loss of weight, cough, infectious process, and hemorrhagic phenomena than their non-coinfected counterparts. Conversely, splenomegaly, hepatomegaly and jaundice were more frequently found in the non-coinfected cases.

Concerning the VL laboratory confirmation, the coinfected cases were mainly diagnosed by a parasitological test, whereas the non-coinfected by an immunological test. Amongst coinfected individuals, the relapse proportion was twice as higher than of non-coinfected. The fatality rate was 25% in coinfected cases, which is three times higher than in non-coinfected.

## Discussion

During the study period, from the total of VL confirmed cases, 760 (2.1%) were coinfected with AIDS (Group 1) and 541 (1.5%) marked as HIV positive in the VL information form (Group 2), were not identified as AIDS cases. The temporal analysis showed an increase in the number of coinfected people during this period, and that this growing tendency, if maintained, will confirm WHO’s expectation that the number of VL/HIV coinfections tends to rise in the next few years [13].

Despite the increase in the number of coinfection cases in Brazil, shown in this study, data from the Brazilian MoH shows that the number of VL cases has remained stable [19]. This observation, together with the finding that coinfected cases come more often from urban areas than non-coinfected cases, suggests that the VL urbanization, which began in the 1980s, contributed to the emergence of VL/HIV coinfection mainly in urban areas of the country.

When we analyzed the spatial distribution of the VL/AIDS coinfection cases, by state, we noticed a record of this disease in all the Brazilian regions, even in states where autochthonous VL cases have not yet been recorded, such as Paraná, Amazonas and Rondônia.

From the total of VL cases in this study, the majority were children under 13 years old (56.49%), which is in line with historical data from the Brazilian MoH, although since 2014 this group accounted for less than 50% of all VL cases in Brazil [20]. The male:female ratio was higher among coinfected patients aged 13 years or over than that found in children under 13 years of age, which was nearly 1:1. Pintado et al [21] also found a higher ratio among coinfected cases; however, this difference was not statistically significant. We also observed that the coinfection cases are higher among adult males, as previously described [21–23].

The average age found among the coinfected cases was 34 years, while, amid the non-coinfected cases it was approximately 19 years, being one of the main differences found between the two groups. Even though young adults were more affected, some authors believe that among the immunocompetent population the disease is more frequent during childhood [21, 23, 24].

In this study, the more frequent clinical manifestations between the coinfected were fever, weakness, weight loss and splenomegaly; some of these results are similar to the ones found by Alexandrino-de-Oliveira [25] and by Pintado et al [21]. On the other hand, some authors affirm that immunodeficient patients present different clinical manifestations, such as lack of visceromegaly or fever [26].

The analyses of the VL clinical manifestations revealed a higher frequency of weakness, loss of weight, cough, infectious process, and hemorrhagic phenomena among the coinfected than in the non-coinfected. Whereas, splenomegaly, hepatomegaly and jaundice were more frequent in non-coinfected cases. The differences found between the two groups were small, however, significant. Although it did not represent the highest proportion amongst coinfected patients, splenomegaly in this group was still elevated (79.7%), which is a similar result found by Nuno-Marques et al [23] and Daher et al [27], but contrary to other studies [28, 29].

The difference between the proportions of splenomegaly among non-coinfected and coinfected was 6.7% (95%CI 4.5 to 9.1), along with coinfected patients presenting a lower frequency of spleen enlargement. These results are similar to the ones found by Fernández-Guerrero et al [30] and Pintado et al [21]; however, in the first study there was no significant difference between patients with or without AIDS. In the second mentioned study, splenomegaly was described as the only VL clinical manifestation with a significant statistical difference among HIV positive and negative individuals, while the results for other typical VL clinical characteristics were similar in the groups, which is the opposite of the results found in the present study. Peters et al [31] described that a lower frequency of splenomegaly among patients coinfected with AIDS is due to a flaw in the macrophages response, seeing that spleen enlargement in VL is related to the macrophage proliferation.

Regarding the VL laboratory confirmation, our results showed that among the coinfected there is a higher positivity in the parasitological diagnosis and lower in the immunological diagnosis, which is similar to the findings of other authors [23, 30–32].

For coinfected individuals, the relapse proportion was twice as higher than of non-coinfected. The tendency to relapse is one of the most observed characteristic in studies of VL with HIV coinfection. Fernández-Guerrero et al [30] described that 40% of the HIV positive individuals had one or more relapses. Alexandrino-de-Oliveira et al [25] reported one or more relapses in 13 coinfected patients from a total of 23 cases, representing 56.5% of relapses.

The fatality rate was 25% in coinfected cases, which is three times higher than in non-coinfected. Several authors have reported a higher fatality rate among VL/HIV and VL/AIDS coinfected patients [21, 23, 31], and the cause of death of coinfected patients being other conditions associated to AIDS, such as opportunistic diseases and therapeutic complications [23]. Nevertheless, Pintado and López-Vélez [32] highlighted the possibility of the VL contribution to death, either by immunosuppression independent of HIV or by stimulation of the HIV replication.

The use of HAART described in a previous study [21], has shown a significant improvement of the survival of *Leishmania*-HIV coinfected patients; however, the reduction of relapses with the use of HAART still deserves further studies. Additionally, the benefit of HAART will not impact the population with difficulties to access the health system, i.e. the impoverished people living in the periphery of the largest cities who ignore their HIV status. Our data demonstrated that 541 VL coinfected patients were not reported in the AIDS database. Then, such underreporting could be explained at least in part by late detection of the HIV infection, considering that most of the symptomatic VL/HIV coinfected patients have lower CD4+ cell counts at the onset of VL symptoms [33].

Considering that the association between VL and AIDS is relatively recent and has been growing worldwide, including in Brazil, this study contributes to the knowledge of profile, magnitude and prognosis of the VL/AIDS coinfection in the country. Moreover, the results may subsidize actions to overcome challenges related to both diseases and assist in guiding actions and decision-making processes.

The increased availability of large databases with computerized health data, in the last decades, has contributed to an ongoing interest of combining records from different databases, in search for complementary information. The present study demonstrates the usefulness of this strategy to reveal the importance of a clinical status with extremely relevant practical implication in the care offered to patients with VL or AIDS.

The epidemiological status of VL/HIV-AIDS coinfection in Brazil shown by this study is concerning. Since a significant portion of the two diseases in Latin America is concentrated in Brazil [3, 34], the growing number of coinfection cases is a challenge for the health system, especially regarding the impact on the fatality rate of VL patients. This behavior can be analyzed as a model of what may occur in other countries where VL has been presenting emerging patterns, such as Paraguay and Argentina.

Another relevant point is the low vulnerability of the VL transmission cycle in face of the current available control interventions, which allows to reasonably affirm that the number of people infected by *L*. *infantum* in Brazil, and other endemic countries of the Latin America, is not going to decrease over the next years. In this regard, interventions aimed at reducing the incidence and early detection of HIV infection, as well as initiating specific antiretroviral treatment, are crucial to reduce the incidence of coinfection in its symptomatic form. Despite it can be argued that the increased coinfection incidence is due to better access to HIV serological testing in VL patients, it would be unlikely to attribute the total phenomenon to the success of this strategy. Since the relationship of the databases identified the existence of a relevant portion of VL patients who were not undergone a serological test to detect HIV infection. Thus, full implementation of HIV testing effectively offered to 100% of VL patients is crucial as stated in the Brazilian MoH recommendations since 2011 [33].

The scenario presented here calls for the urgent need to implement efficient policies that allow early detection and treatment of HIV infection, before VL development, to avoid symptomatic cases of coinfection. Once the symptomatic VL/HIV coinfection is present it would be essential to maintain reference centers with high clinical and technological skills to offer proper care, including effective and less toxic drugs, and follow-up to achieve a lower case-fatality rate.

Finally, the improvement of specific VL treatment for HIV coinfected people deserves especial attention because of the poor prognosis related to the toxicity of the currently available drugs, mainly meglumine antimoniate and amphotericin B deoxycholate. Despite access of coinfected patients to liposomal amphotericin B being universal and free of charge in Brazil and the fact that this drug represents a significant advance to toxicity reduction, there is a lack of long-term efficacy, seeing the occurrence of relapses, which needs to be properly addressed [35]. Combination therapy and optimized secondary prophylaxis must be considered; furthermore, carefully designed clinical trials and observational studies must be prioritized in the near future.

## Supporting information

S1 Table Demographic, clinical and epidemiological characteristics of children under 13 years of age according to the analyzed group. Brazil. 2001–2010. (DOCX)

S2 Table Demographic, clinical and epidemiological characteristics of patients aged 13 years or over, according to the analyzed group. Brazil. 2001–2010. (DOCX)
