# Supplementary material for: Visceral leishmaniasis and HIV/AIDS in Brazil: Are we aware enough?
Source: PLoS Negl Trop Dis. 2017 Sep 25;11(9):e0005772. doi: 10.1371/journal.pntd.0005772 (PMC5612457; doi:10.1371/journal.pntd.0005772)
Supplement: S1 Table — (DOCX) [file pntd.0005772.s001.docx]

**S1 Table. Demographic, clinical and epidemiological characteristics of children under 13 years of age according to the analyzed group. Brazil. 2001-2010.**

| Characteristics | Group 1  (VL/AIDS coinfected) | | | Group 2  (VL/HIV coinfected) | | Group 3  (Non-coinfected) | |
| --- | --- | --- | --- | --- | --- | --- | --- |
|  | Frequency (total^*^) | | % | Frequency (total^*^) | % | Frequency (total^*^) | % |
| Age |  | | | | | | |
| average | 5.16 | | | 3.9 | | 3.77 | |
| median | 4 | | | 2 | | 3 | |
| standard deviation | 4.04 | | | 3.57 | | 3.07 | |
| Gender |  | | | | | | |
| male | 20 (33) | | 60.6 | 64 (125) | 51.2 | 10513 (20074) | 52.4 |
| female | 13 (33) | | 39.4 | 61 (125) | 48.8 | 9561 (20074) | 47.6 |
| Ethnic Group/Race |  | | | | | | |
| white | 6 (27) | | 22.2 | 19 (95) | 20.0 | 3455 (16053) | 21.5 |
| black | 5 (27) | | 18.5 | 7 (95) | 7.4 | 1084 (16053) | 6.8 |
| yellow | 0 (27) | | 0.0 | 2 (95) | 2.1 | 164 (16053) | 1.0 |
| mixed ("parda") | 16 (27) | | 59.3 | 67 (95) | 70.5 | 11194 (16053) | 69.7 |
| indigenous | 0 (27) | | 0.0 | 0 (95) | 0.0 | 156 (16053) | 1.0 |
| Residency area |  | | | | | | |
| urban/peri-urban | 26 (32) | | 81.2 | 85 (117) | 72.6 | 13157 (19454) | 67.6 |
| rural | 6 (32) | | 18.8 | 32 (117) | 27.4 | 6297 (19454) | 32.4 |
| Clinical manifestation |  | | | | | | |
| fever | 31 (31) | | 100.0 | 119 (122) | 97.5 | 18831 (19331) | 97.4 |
| weakness | 23 (29) | | 79.3 | 77 (102) | 75.5 | 13860 (17673) | 78.6 |
| weight loss | 23 (28) | | 82.1 | 82 (109) | 75.2 | 12651 (17733) | 71.3 |
| cough | 15 (29) | | 51.7 | 51 (101) | 50.5 | 8882 (17434) | 50.9 |
| splenomegaly | 26 (31) | | 83.9 | 106 (117) | 90.6 | 17133 (19039) | 90.0 |
| hepatomegaly | 24 (30) | | 80.0 | 94 (112) | 83.9 | 15056 (18765) | 80.2 |
| edema** | 3 (15) | | 20.0 | 14 (58) | 24.1 | 1744 (6921) | 25.2 |
| pallor** | 10 (14) | | 71.4 | 51 (61) | 83.6 | 5607 (7037) | 79.7 |
| infectious process** | 7 (15) | | 46.7 | 17 (58) | 29.3 | 1600 (6746) | 24.6 |
| hemorrhagic phenomena** | 2 (15) | | 13.3 | 6 (58) | 10.3 | 490 (6860) | 7.1 |
| jaundice** | 1 (15) | | 6.7 | 20 (58) | 34.5 | 1499 (6886) | 21.8 |
| Parasitological diagnosis |  | | | | | | |
| positive | 16 (31) | | 51.6 | 50 (120) | 41.6 | 7798 (18292) | 42.6 |
| negative | 2 (31) | | 6.5 | 8 (120) | 6.7 | 1864 (18292) | 10.2 |
| unperformed | 13 (31) | | 41.9 | 62 (120) | 51.7 | 8630 (18292) | 47.2 |
| Immunological diagnosis (IFA) |  | | | | | | |
| positive | 19 (30) | | 63.4 | 58 (116) | 50.0 | 8436 (17427) | 48.4 |
| negative | 1 (30) | | 3.3 | 10 (116) | 8.6 | 1447 (17427) | 8.3 |
| unperformed | 10 (30) | | 33.3 | 48 (116) | 41.4 | 7544 (17427) | 43.3 |
| Patient entry |  | | | | | | |
| new case | 31 (32) | | 96.9 | 118 (122) | 96.7 | 18437 (19017) | 97.0 |
| relapse | 1 (32) | | 3.1 | 4 (122) | 3.3 | 580 (19017) | 3.0 |
| Initial drug administrated |  | | | | | | |
| pentavalent antimony | 27 (31) | 87.0 | | 88 (112) | 78.6 | 15708 (17889) | 87.8 |
| amphotericin b | 2 (31) | 6.5 | | 9 (112) | 8.1 | 1142 (17889) | 6.4 |
| pentamidine | 0 (31) | 0.0 | | 1 (112) | 0.9 | 88 (17889) | 0.5 |
| other | 0 (31) | 0.0 | | 7 (112) | 6.2 | 506 (17889) | 2.8 |
| not used | 2 (31) | 6.5 | | 7 (112) | 6.2 | 445 (17889) | 2.5 |
| liposomal amphotericin b | 0 (15) | 0.0 | | 7 (59) | 11.9 | 416 (6932) | 6.0 |
| Progression |  | | | | | | |
| recovery | 22 (26) | 84.6 | | 87 (103) | 84.5 | 15595 (17138) | 91.0 |
| death | 3 (26) | 11.5 | | 12 (103) | 11.6 | 864 (17138) | 5.0 |
| abandonment/transference | 1 (26) | 3.9 | | 4 (103) | 3.9 | 679 (17138) | 4.0 |
| Confirmation criterion** |  | | | | | | |
| laboratory | 15 (15) | 100.0 | | 49 (60) | 81.7 | 6361 (7651) | 83.1 |
| clinical epidemiological | 0 (15) | 0.0 | | 11 (60) | 18.3 | 1290 (7651) | 16.9 |
| * Numbers may vary due to missing values for some variables  ** Variables available only in the VL database from 2007-2010. | | | | | | | |
