# Supplementary material for: Visceral leishmaniasis and HIV/AIDS in Brazil: Are we aware enough?
Source: PLoS Negl Trop Dis. 2017 Sep 25;11(9):e0005772. doi: 10.1371/journal.pntd.0005772 (PMC5612457; doi:10.1371/journal.pntd.0005772)
Supplement: S2 Table — (DOCX) [file pntd.0005772.s002.docx]

**S2 Table.** **Demographic, clinical and epidemiological characteristics of patients aged 13 years or over, according to the analyzed group. Brazil. 2001-2010**.

| Characteristics | Group 1  VL/AIDS coinfected | | Group 2  VL/HIV coinfected | | Group 3  Non-coinfected | |
| --- | --- | --- | --- | --- | --- | --- |
|  | **Frequency (total*)** | **%** | **Frequency (total*)** | **%** | **Frequency (total*)** | **%** |
| Age |  | | | | | |
| average | 37.97 | | 38.58 | | 39.52 | |
| median | 37 | | 36 | | 34 | |
| standard deviation | 10.37 | | 14.96 | | 22.66 | |
| Gender |  | | | | | |
| male | 558 (727) | 76.8 | 331 (416) | 79.6 | 10365 (14441) | 71.8 |
| female | 169 (727) | 23.2 | 85 (416) | 20.4 | 4076 (14441) | 28.2 |
| Ethnic Group/Race |  | | | | | |
| white | 141 (622) | 22.7 | 83 (349) | 23.8 | 2387 (11443) | 20.9 |
| black | 63(622) | 10.2 | 33 (349) | 9.5 | 1386 (11443) | 12.1 |
| yellow | 4 (622) | 0.6 | 4 (349) | 1.1 | 148 (11443) | 1.3 |
| mixed ("parda") | 412 (622) | 66.2 | 228 (349) | 65.3 | 7460 (11443) | 65.2 |
| indigenous | 2 (622) | 0.3 | 1 (349) | 0.3 | 62 (11443) | 0.5 |
| Residency area |  | | | | | |
| urban/peri-urban | 626 (704) | 88.9 | 359 (397) | 90.4 | 10485 (13946) | 75.2 |
| rural | 78 (704) | 11.1 | 38 (397) | 9.6 | 3461 (13946) | 24.8 |
| Clinical manifestation |  | | | | | |
| fever | 636 (696) | 91.4 | 365 (403) | 90.6 | 12716 (13649) | 93.2 |
| weakness | 608 (678) | 89.7 | 343 (394) | 87.1 | 11265 (12831) | 87.8 |
| weight loss | 596 (682) | 87.4 | 340 (396) | 85.9 | 10643 (12820) | 83.0 |
| cough | 414 (666) | 62.2 | 230 (382) | 60.2 | 5919 (12370) | 47.8 |
| splenomegaly | 512 (655) | 78.2 | 307 (390) | 78.7 | 10807 (13291) | 81.3 |
| hepatomegaly | 473 (652) | 72.5 | 273 (391) | 69.8 | 9363 (13155) | 71.2 |
| edema** | 88 (341) | 25.8 | 67 (260) | 25.8 | 1411 (5546) | 25.4 |
| pallor** | 265 (346) | 76.6 | 185 (264) | 70.1 | 3957 (5626) | 70.3 |
| infectious process** | 136 (330) | 41.2 | 106 (257) | 41.2 | 1236 (5363) | 23.0 |
| hemorrhagic phenomena** | 46 (339) | 13.6 | 51 (252) | 20.2 | 756 (5507) | 13.7 |
| jaundice** | 57 (335) | 17.0 | 52 (252) | 20.6 | 1521 (5515) | 27.6 |
| Parasitological diagnosis |  | | | | | |
| positive | 372 (679) | 54.8 | 251 (408) | 61.5 | 5937 (13242) | 44.8 |
| negative | 93 (679) | 13.7 | 39 (408) | 9.6 | 1552 (13242) | 11.7 |
| unperformed | 214 (679) | 31.5 | 118 (408) | 28.9 | 5753 (13242) | 43.5 |
| Immunological diagnosis (IFA) |  | | | | | |
| positive | 265 (641) | 41.3 | 142 (393) | 36.1 | 6188 (12739) | 48.6 |
| negative | 70 (641) | 10.9 | 42 (393) | 10.7 | 832 (12739) | 6.5 |
| unperformed | 306 (641) | 47.8 | 209 (393) | 53.2 | 5719 (12739) | 44.9 |
| Patient entry |  | | | | | |
| new case | 636 (677) | 93.9 | 368 (399) | 92.2 | 13203 (13619) | 96.9 |
| relapse | 41 (677) | 6.1 | 31 (399) | 7.8 | 416 (13619) | 3.1 |
| Initial rug administrated |  | | | | | |
| pentavalent antimony | 402 (619) | 64.9 | 210 (355) | 59.1 | 10540 (12601) | 83.6 |
| amphotericin b | 176 (619) | 28.4 | 110 (355) | 31.0 | 1069 (12601) | 8.5 |
| pentamidine | 0 (619) | 0.0 | 2 (355) | 0.6 | 55 (12601) | 0.4 |
| other | 12 (619) | 2.0 | 15 (355) | 4.2 | 374 (12601) | 3.0 |
| not used | 29 (619) | 4.7 | 18 (355) | 5.1 | 563 (12601) | 4.5 |
| liposomal amphotericin b | 48 (348) | 13.8 | 45 (275) | 16.4 | 438 (5580) | 7.8 |
| Progression |  | | | | | |
| recovery | 429 (625) | 68.6 | 266 (359) | 74.1 | 10412 (12296) | 84.7 |
| death | 175 (625) | 28.0 | 78 (359) | 21.7 | 1487 (12296) | 12.1 |
| abandonment/transference | 21 (625) | 3.4 | 15 (359) | 4.2 | 397 (12296) | 3.2 |
| Confirmation criterion** |  | | | | | |
| laboratory | 338 (390) | 86.7 | 255 (282) | 90.4 | 5410 (6196) | 87.3 |
| clinical epidemiological | 52 (390) | 13.3 | 27 (282) | 9.6 | 786 (6196) | 12.7 |
| * Numbers may vary due to missing values for some variables  ** Variables available only in the VL database from 2007-2010. | | | | | | |
